# Toronto aortic stenosis quality of life questionnaire (TASQ): validation in TAVI patients

**DOI:** 10.1186/s12872-020-01477-2

**Published:** 2020-05-05

**Authors:** Rima Styra, Michelle Dimas, Kathy Svitak, Mamta Kapoor, Mark Osten, Maral Ouzounian, Gerald Devins, Amy Deckert, Eric Horlick

**Affiliations:** 1 Center for Mental Health, University Health Network, Toronto, Ontario Canada; 2 Peter Munk Cardiac Center, University Health Network, Toronto, Ontario Canada

**Keywords:** Aortic stenosis, TAVI, Quality of life, Patient-reported outcome measurement

## Abstract

**Background:**

Aortic stenosis (AS) is a common cardiac condition whose prevalence increases with age. The symptom burden associated with severe aortic stenosis (AS) can introduce significant lifestyle disruptions and if left untreated can lead to a poor prognosis. Quality of life (QoL) is an important consideration in these patients. The TASQ is a QoL tool that was developed for aortic stenosis patients. We evaluated the psychometric properties of this specific questionnaire in patients who underwent transcatheter aortic valve implantation (TAVI), which is a therapeutic option for patients with severe aortic stenosis (AS).

**Methods:**

The properties of the TASQ in measuring QoL were evaluated in AS patients undergoing TAVI. Patients presenting for the TAVI procedure (*N* = 62) were evaluated pre-TAVI, at discharge, 1-month, and 3-month follow-ups. Demographic information as well as caregiver status, and daily activities were recorded. In addition to the TASQ, they completed the KCCQ (Kansas City Cardiomyopathy Questionnaire) and the IIRS (Illness Intrusiveness Rating Scale).

**Results:**

The TASQ is a 16-item self-administered questionnaire that assesses AS-specific QoL across five domains: physical symptoms; physical limitations; emotional impact; social limitations, and health expectations. TASQ subscales are internally consistent (α = 0.74–0.96) and showed significant improvements from baseline across assessments (*p* < 0.001). Construct validity evidence was demonstrated by correlations consistent with theoretically derived hypotheses across time points.

**Conclusions:**

The TASQ is a brief measure of AS-specific QoL that is sensitive to change in patients undergoing TAVI. Items on the TASQ capture important QoL concerns reported by AS patients, suggesting this is a measure of relevant and meaningful outcomes for this patient population. Detection of early improvements in QoL by the TASQ is promising, with important implications for the evaluation of procedural outcomes in this population.

## Background

Symptom burden associated with severe aortic stenosis (AS) can introduce significant lifestyle disruptions, particularly by interfering with the capacity to engage in valued activities, interests, and relationships, which can, of course, compromise quality of life (QoL) [1, 2]. Transcatheter aortic valve implantation (TAVI) is indicated for symptomatic patients with severe AS to alleviate symptom burden and to prolong life [3, 4]. Patients undergoing TAVI are considered higher risk for surgery due to their advanced age and multiple comorbidities [5–8]. Understanding the overall effect of illness on a patient’s life is fundamental to planning treatment that optimizes symptom management and satisfaction with outcomes [9, 10]. Canadian guidelines for the quality of TAVI care underscore the importance of documenting patient-reported outcome measures (PROM) to understand the patient perspective particularly with respect to QoL [11].

Presently, approximately 180,000 patients annually can be considered potential TAVI candidates in the European Union and North America [12]. This increasing number of TAVI patients will have an impact on healthcare planning and it is imperative that policy makers understand the variables that determine QoL in patients with AS. This can allow for a broader focus not only on symptom reduction, but a more holistic approach to patient-identified recovery and QoL [13, 14]. Formal QoL assessment by clinicians can improve patient-physician communication, clinical decision-making, and satisfaction with care [15].

QoL scales implemented in cardiac care assess QoL using generic measures e.g., EuroQoL five dimensions questionnaire (EQ-5D) [16], the Short Form Health Survey-36 (SF-36) [17], and its abbreviated form, the SF-12 [18]. Disease-specific scales are also used to assess QoL, such as QoLmeasures for patients with heart failure which are widely used in the cardiac care setting (e.g., Kansas City Cardiomyopathy Questionnaire (KCCQ) [19], and the Minnesota Living with Heart Failure Questionnaire (MHLF)) [20]. Generic instruments are especially useful because they can compare QoL across patient populations by providing a common metric. They do not, however, address issues or effects that are specific to a given population (e.g., aortic stenosis). There is currently no AS-specific measure of QoL.

Clinicians require a framework to understand and to evaluate the effects of disease and interventions from a patient perspective. Physical domains cannot capture the entire picture because emotional domains are central to QoL and do not map isomorphically onto the physical domains. Identifying factors that impact QoL are fundamental to identifying and addressing patients’ psychosocial and functional needs. In developing the TASQ, we focused on ensuring patient involvement and its development has previously been reviewed [21].

We undertook the evaluation of the new instrument’s properties evaluating QoL in patients with AS who underwent a TAVI. We administered the TASQ to a sample of AS patients undergoing TAVI to evaluate its properties.

## Methods

The study was carried out in a tertiary care urban center which includes a center of excellence in cardiac care. The Research Ethics Board reviewed and approved the protocol for the study.

Patients meeting cardiac criteria for the TAVI procedure, fluent in English, 18 years and older, who were identified during a standard clinical visit were eligible to participate in the study. Patients completed the assessment package which included the questionnaires in paper format at clinic visits pre-TAVI, with follow-up assessments at time of discharge, 1-month, and 3-months. Participant sociodemographic characteristics collected included age, sex, marital status, living arrangements, and activities of daily living. To assess the construct validity of the TASQ, participants were asked to complete in addition to the TASQ, two other measures – the Kansas City Cardiomyopathy Questionnaire (KCCQ) and the Illness Intrusiveness Rating Scale (IIRS) to assess overall QoL at four time points.

The TASQ [22] is a 16-item self-administered questionnaire that requires approximately 5 min to complete. There are four derived subscales: physical symptoms (2 items); physical limitations (4 items); emotional impact (7 items); and social limitations (2 items). A single item taps health expectations. For each item, participants are asked to rate the current interference of AS on a 7-point scale anchored by “not very much” to “very much”. Items are reverse-coded and summed to compute a total score. Subscale scores may be calculated by summing the reverse-coded items within the subscale. Scores may range from 16 to 112. Higher scores reflect greater perceived QoL. Each of the domains can be scored seperately by generating the sum of constituent item responses.

The KCCQ is a 15-item self-report measure of health status for patients with heart failure [19]. The KCCQ measures five domains - physical limitations, symptoms, self-efficacy, social interference and QoL. This questionnaire has been widely used in the TAVI patient population [22–24]. Scores range from 0 to 100, with high scores representing high QoL. Illness intrusiveness was assessed using the Illness Intrusiveness Rating Scale (IIRS), a 13-item self-report measure of the extent to which disease and/or treatment interfere with psychologically meaningful activities in important life domains [25]. The IIRS has been extensively used in chronic disease populations and has strong psychometric characteristics, including its responsiveness to change following therapeutic intervention [25]. Scores range from 13 to 31, with high scores representing high levels of illness intrusiveness.

### Statistical analysis

We calculated descriptive statistics (means, standard deviation, percentages) for all demographic variables, such as age, sex, marital status, living arrangements, caregiver role, and ADLs, as well as the scores for the TASQ, KCCQ and the IIRS. TASQ item mean, standard deviation, range, skewness and kurtosis at baseline were calculated. Cronbach’s alpha indicated the internal consistency of the TASQ at each assessment and for each domain and the inter-item correlation matrix. Our a-priori criterion for internal consistency was a Cronbach’s alpha of ≥0.70 [26]. Separate Pearson correlation coefficients summarized the relations between each TASQ domain and the KCCQ and the IIRS to evaluate construct validity. We hypothesized a priori that the TASQ physical symptoms and physical limitations domains would correlate: (a) positively with the KCCQ symptom related and QoL domains, and (b) negatively with the IIRS instrumental subscale. We further hypothesized that: (a) the TASQ emotional impact, social limitations, and health expectations domains would correlate positively with the KCCQ QoL and social interference domains, and (b) negatively with the IIRS relationships and personal development subscales.

## Results

Of the eligible patients, 62 participants completed > 1 follow-up assessment and were included in statistical analyses. One participant was an age-related outlier (age = 48 years, which was 22 years younger than the next youngest respondent) and was thus excluded from the analysis as an age-related outlier. Thirty day mortality rate was 3% - 2 patients expired after discharge from hospital. One patient developed delirium related to a lung infection. No patients suffered a cerebral vascular accident (CVA). All participants completed the questionnaire package at pre-TAVI and at discharge (100%), 1 month (81%) and 3 months (69%). The majority of patients were lost towards the end of the study, with 9% of patients being lost to followup and 14% not returning questionnaires at 3 months.

Participants were predominantly male (64.5%), married or partnered (54.8%), and the majority (82.5%) lived with either their spouse or family members. The majority of participants were independent in their activities of daily living (72.5%) and eight (12.9%) identified as the primary caregiver for a loved one (Table 1).

**Table 1:** Participant characteristics at baseline (*N* = 62)

Variable	No. of patients	
Age (years)	62	83.45 ± 5.45
Sex		
Male	40	64.5%
Marital status		
Married/common law partner	34	54.8%
Divorced	3	4.8%
Widowed	23	37.1%
Single	2	3.2%
Living arrangements		
Living alone	15	24.2%
Living with spouse/partner	29	46.7%
Living with family	16	25.8%
Living in retirement or long-term care facility	2	3.2%
Caregiver role	8	12.9%
Activities of daily living*		
Completely independent	45	72.5%
Needs help with physical chores	14	22.6%

*Three participants with a missing value

### Scale characteristics and internal consistency

Item characteristics at baseline (pre-TAVI), including means, standard deviations, skewness and kurtosis are presented in Table 2. The TASQ domain scores and total score demonstrated good to excellent internal consistency at all timepoints; with the exception of the follow-up assessments of the physical symptom domain (Cronbach’s alphas reported in Table 3). We did not calculate Cronbach’s alpha for health expectations because it involves a single item. Correlations between subscales were mostly moderate to high (Table 4).

**Table 2:** TASQ item characteristics at baseline (pre-TAVI)

Item	Mean ± SD	Range	Skewness	Kurtosis
**Physical symptoms**	7.32 **±** 3.03	2–13	0.21	− 0.87
Shortness of breath	3.87 **±** 2.05	1–7	0.24	−1.15
Ratings of overall health	3.45 **±** 1.25	1–6	0.22	− 0.38
**Physical limitations**	13.08 **±** 6.31	4–24	0.12	−1.13
Heart problems interfering with doing daily chores	3.76 **±** 2.18	1–7	0.14	−1.3
Heart problems interfering with being able to walk without resting	3.21 **±** 2.05	1–7	0.58	−1.02
Shortness of breath or extreme tiredness when exercising	2.81 **±** 2.02	1–7	1.01	−0.25
Ratings of ability to do things	3.31 **±** 1.47	1–7	0.38	0.85
**Emotional impact**	30.24 **±** 11.14	8–48	− 0.18	− 0.95
Worried about having a heart attack or dying	5.02 **±** 2.03	1–7	− 0.75	− 0.73
Frustrated about having to stay or go to the hospital because of heart problems	4.29 **±** 2.36	1–7	− 0.24	−1.54
Feeling discouraged about being very tired	3.89 **±** 2.14	1–7	0.18	−1.38
Worried about what will happen to your family if you don’t get better	4.18 **±** 2.35	1–7	−0.16	−1.56
Worried about what will happen financially	5.58 **±** 2.00	1–7	−1.20	0.07
Feeling unable to make plans for the future	4.05 **±** 2.22	1–7	− 0.04	−1.51
Enjoyment of life limited by health problems	3.24 **±** 2.06	1–7	0.53	−1.05
**Social Limitations**	8.82 **±** 4.32	2–14	−0.16	−1.38
Heart problems interfering with going out with friends or to social events	4.34 **±** 2.19	1–7	−0.11	−1.39
Heart problems interfering with going out to visit family	4.48 **±** 2.22	1–7	− 0.28	−1.35
**Health Expectations**				
Rating of hope for health improvements	2.48 **±** 1.53	1–7	0.96	0.62
**TASQ Total score**	61.85 **±** 21.97	23–106	0.10	− 0.85

**Table 3:** Internal Consistency (Coefficient Alpha) TASQ Subscales Across Four Measurement Occasions

Toronto Aortic Stenosis Quality of Life Scale	Pre-TAVI (baseline)	Discharge	1-month	3-month
Physical Symptoms	0.75	0.66	0.53	0.58
Physical Limitations	0.82	0.86	0.86	0.83
Emotional Impact	0.86	0.84	0.84	0.84
Social Limitations	0.96	0.95	0.91	0.93
Total score	0.92	0.91	0.92	0.89

**Table 4:** TASQ Inter-item Correlation Matrix

TASQ Subscales	Physical Symptoms	Physical Limitations	Emotional Impact	Social Limitations
Physical Symptoms	1.00			
Physical Limitations	0.76	1.00		
Emotional Impact	0.59	0.67	1.00	
Social Limitations	0.59	0.74	0.56	1.00

### Responsiveness

**Fig. 1:**
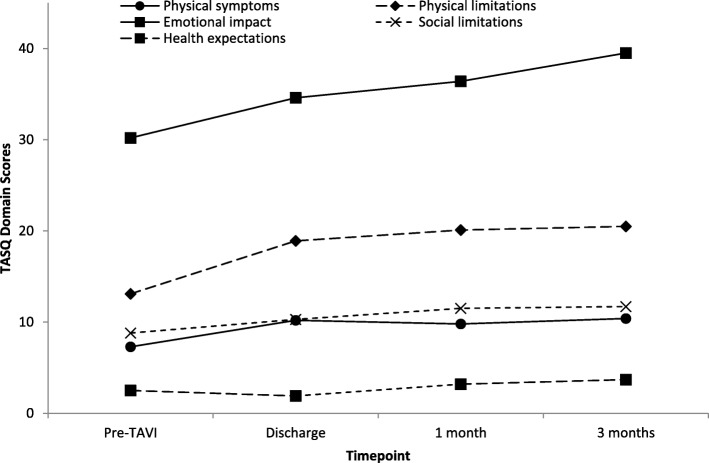
TASQ scores by domain over time

The TASQ demonstrated sensitivity to change from baseline to each of the three measurement occasions. Total TASQ scores improved significantly from baseline to all three occasions: (a) discharge (*p* < 0.0001, *d* = 0.92); (b) 1-month (*p* < 0.0001, *d* = 0.94); and (c) 3-month (*p* < 0.0001, *d* = 0.99) follow-up assessments. All domain scores improved significantly over time (*p*’s < 0.03), with the one exception of the 3-month follow-up assessment of health expectations (*p* < 0.10). Figure 1 plots changes over time in TASQ domain scores from baseline to 3 months post-TAVI.

### Construct validity

As hypothesized, the TASQ physical symptoms and physical limitations domains correlated significantly with the KCCQ symptom-related and QoL domains, and with the IIRS instrumental subscale. Symptom-related correlations tended to be consistent across assessments. Emotional impact and social limitations correlated significantly and as hypothesized with the KCCQ social interference, and QoL domains and with the IIRS relationships and personal development subscale. Significant correlations at baseline were maintained across timepoints, despite overall improvement in TASQ scores. The health expectations domain did not correlate significantly with the KCCQ or the IIRS. Tables 5, 6, 7 and 8 report correlations and the valence of all correlation coefficient’s calculated within each of four separate measurement occasions - pre-TAVI, at discharge, 1-month, and at 3 month measurements.

**Table 5:** Construct Validity: Pearson Correlation Coefficients between variables pre-TAVI (T0)

	TASQ Physical Symptoms	TASQ Physical Limitations	TASQ Emotional Impact	TASQ Social Limitations	TASQ Health Expectations
KCCQ Physical Limitation	0.65**	0.69**	0.42**	0.57**	−0.29
KCCQ Symptom Stability	0.33**	0.42**	0.40**	0.11	− 0.57
KCCQ Symptom Frequency	0.75**	0.70**	0.57**	0.56**	0.18
KCCQ Symptom Burden	0.73**	0.70**	0.64**	0.50**	0.15
KCCQ Self-Efficacy	0.28*	0.38**	0.27*	0.23	−0.06
KCCQ Quality of Life	0.69**	0.68**	0.71**	0.49**	0.30*
KCCQ Social Limitation	0.56**	0.62**	0.46**	0.58**	0.20
IIRS Instrumental	−0.35**	− 0.40**	− 0.38**	− 0.32*	−0.10
IIRS Relationships and Personal Development	−0.43**	− 0.54**	− 0.45**	− 0.51**	− 0.08
IIRS Intimacy	−0.28*	− 0.27*	− 0.31*	− 0.26*	−0.14

*Note.* ** *p* < 0.01; *p < 0.05

**Table 6:** Construct Validity: Pearson Correlation Coefficients between variables at discharge (T1)

	TASQ Physical Symptoms	TASQ Physical Limitations	TASQ Emotional Impact	TASQ Social Limitations	TASQ Health Expectations
KCCQ Physical Limitation	0.50**	0.58**	0.38**	0.48**	−0.34*
KCCQ Symptom Stability	0.38**	0.49**	0.16	0.29*	−0.19
KCCQ Symptom Frequency	0.50**	0.48**	0.43**	0.42**	0.03
KCCQ Symptom Burden	0.58**	0.59**	0.60**	0.56**	0.12
KCCQ Self-Efficacy	0.31*	0.25*	0.22	0.09	0.01
KCCQ Quality of Life	0.62**	0.64**	0.63**	0.52**	0.07
KCCQ Social Limitation	0.40**	0.46**	0.41**	0.41**	−0.14
IIRS Instrumental	−0.49**	− 0.59**	− 0.62**	− 0.51**	0.01
IIRS Relationships and Personal Development	− 0.34**	− 0.54**	− 0.46**	−0.61**	− 0.03
IIRS Intimacy	−0.19	− 0.30*	−0.40**	− 0.20	−0.14

*Note.* **p < 0.01; * *p* < 0.05

**Table 7:** Construct Validity: Pearson Correlation Coefficients between variables at 1-month follow-up (T2)

	TASQ Physical Symptoms	TASQ Physical Limitations	TASQ Emotional Impact	TASQ Social Limitations	TASQ Health Expectations
KCCQ Physical Limitation	0.38**	0.53**	0.44**	0.41**	−0.09
KCCQ Symptom Stability	−0.25	0.01	0.05	0.02	−0.19
KCCQ Symptom Frequency	0.41**	0.44**	0.56**	0.14	0.01
KCCQ Symptom Burden	0.55**	0.65**	0.57**	0.25	−0.07
KCCQ Self-Efficacy	0.48**	0.40**	0.32*	0.07	−0.01
KCCQ Quality of Life	0.51**	0.57**	0.55**	0.47**	−0.16
KCCQ Social Limitation	0.53**	0.70**	0.59**	0.67**	−0.02
IIRS Instrumental	−0.51**	−0.58**	− 0.60**	−0.53**	0.06
IIRS Relationships and Personal Development	−0.46**	−0.52**	− 0.48**	−0.38*	− 0.08
IIRS Intimacy	−0.09	− 0.08	−0.18	− 0.07	0.14

*Note.* **p < 0.01; *p < 0.05

**Table 8:** Construct Validity: Pearson Correlation Coefficients between variables at 3-month follow-up (T3)

	TASQ Physical Symptoms	TASQ Physical Limitations	TASQ Emotional Impact	TASQ Social Limitations	TASQ Health Expectations
KCCQ Physical Limitation	0.27	0.61**	0.52**	0.54**	−0.07
KCCQ Symptom Stability	0.09	−0.20	−0.16	− 0.06	−0.12
KCCQ Symptom Frequency	0.75**	0.57**	0.50**	0.65**	−0.36*
KCCQ Symptom Burden	0.64**	0.80**	0.56**	0.81**	−0.21
KCCQ Self-Efficacy	0.36*	0.26	0.17	0.28	−0.05
KCCQ Quality of Life	0.62**	0.55**	0.60**	0.64**	−0.39
KCCQ Social Limitation	0.65**	0.45*	0.55**	0.58**	−0.34
IIRS Instrumental	−0.36*	−0.61**	− 0.51**	−0.70**	− 0.49
IIRS Relationships and Personal Development	−0.40*	− 0.52**	−0.43*	− 0.66**	0.22
IIRS Intimacy	−0.18	−0.39*	− 0.14	−0.35	0.07

*Note.* **p < 0.01; *p < 0.05

## Discussion

The goal of the TASQ is to provide another option to measure QoL for AS patients based on issues that this patient population identified as significant to them. This questionnaire is focused on the AS population which is different from other questionnaires such as the SF-36, EQ-5D, the KCCQ and the MHLF.

The TASQ was developed based on a sizable cohort of patients to ascertain the impact of AS on QoL. This instrument involved extensive patient input (*N* = 333) to explore the impact on various strategic areas of patients’ lives that form the basis of a person’s QoL [21]. The TASQ is grounded in the principle that what may be regarded as a good outcome by a clinician may differ from what is regarded as important to the patient. The person-centred perspective implicit in the TASQ highlights the aspects of QoL that are most important to patients with AS, and assists in documenting the substantial emotional impact they experience as compared to other scales. It offers valuable insight with regards to how patients are impacted and cope with this condition on a daily basis.

TASQ subscales tap meaningful outcomes for this patient population and the instrument is relevant and sensitive to changes in QoL that occur following therapeutic intervention. The TASQ is easy to administer and score. It may thus be useful clinically as a PRO measure to monitor QoL in patients with AS, both before and after intervention. Our findings suggest that the TASQ possesses strong psychometric properties and provides insight into the QoL that patients experience.

A strength of the TASQ is that it does not focus predominantly on physical symptoms or treatment issues but takes into account the social and emotional aspects of QoL, thus incorporating a comprehensive approach to QoL. The TASQ is a comprehensive tool for QoL assessment as well as a potential tool for assisting the evaluation process for clinicians [21]. It provides the patient with a tool to inform the clinician of what is important to them. The TASQ can support patient-clinician communication and consideration of ongoing care options. It has been raised that decision making regarding the appropriateness of an aortic valve replacement should include an assessment of the potential effects on overall QoL rather than solely focusing on physical cardiac symptoms [27].

The TASQ offers a novel contribution to existing assessments of QoL in patients with AS. Items and domain areas are relevant to the target population; derived from concerns expressed by a large cohort of patients with AS being assessed. The development of the TASQ incorporated our understanding of the types of activities that patients consider meaningful and captures relevant QoL concerns and expectations. An important element of patient-centered care is that clinical evaluations of disease and therapeutic interventions be supplemented by patient expectations and subjective experiences [28]. Such person-centred criteria for QoL in patients with AS include the ability to engage in personally meaningful activities, such as returning to work or spending time with grandchildren.

Our findings supported the reliability and construct validity of the TASQ four measurement occasions that represent meaningful clinical milestones. We detected significant improvements in the TASQ overall and in domain scores at all three follow-up occasions, demonstrating that the TASQ is responsive to changes in QoL in TAVI patients. Notably, the TASQ detected significant improvements in QoL at the time of discharge. These findings are consistent with TAVI outcome literature indicating significant recovery to left ventricular ejection fraction in 62% of patients before discharge with little change from 48 hr (65.7%) to 1 year post TAVI (70.0%) [29]. Age may be less of a factor in terms of improvement since early and long term improvement in QoL has been shown for elderly TAVI patients in comparison to younger patients [30, 31]. However, among elderly patients, especially with multiple comorbidities, the survival benefit may be less sizable, and this increases the importance of using a more holistic measure of overall QoL [32]. The identification of early improvement may be important to a variety of patients; for example, those expressing a need to return to work, those who declared themselves the primary caregiver for a spouse or child, and older deconditioned-patients requiring improvement in stamina to undergo rehabilitation or proceeding to further surgical procedures that were postponed because of the cardiac issues.

The TASQ is a brief self-administered questionnaire with visually clear responses. It is simple and brief so that it can be incorporated effectively into busy clinical practices. Researchers have noted in an older population difficulty when completing questionnaires which are lengthy or difficult to comprehend [30]. It offers valuable insight with regards to how patients are impacted and cope with this condition on a daily basis. The development and validation of the TASQ supports its use in the evaluation of QoL in patients.

### Limitations

The psychometric properties of the TASQ were assessed using a convenience sample of patients with AS being considered for TAVI at a single, major urban cardiac centre. Future research should include comparison groups (e.g. SAVR) to further our understanding of QoL in AS and to inform TAVI patient expectations and treatment decision-making. Future psychometric studies of the TASQ questionnaire should aim for a larger sample size to contribute to our understanding of the measurement properties. Moreover, administration with a larger sample size would further inform the usefulness of the TASQ questionnaire in routine clinical care. It is not surprising that we detected a post-procedural decline in the internal consistency of the TASQ physical symptoms subscale because the items comprising this subscale are differentially impacted by TAVI, which reduces their interrelatedness over time. Shortness of breath is a burdensome symptom alleviated by TAVI (item 1); however, post-procedural subjective ratings of heart health (item 14) often differ when patients’ perspectives are compared to those of cardiac-care providers.

A concern is that the robustness of the data at 3 months may have been impacted by the loss of participants at the 3 month followup. The majority of fall-off of patient responses occurred at 3 months, and we were unable to enquire as to the reason. We anticipate that given the older overall age of our participants, there may have been some study fatigue. The high rates of completion pre-TAVI, pre-discharge (100%) and at 1 month (81%), provided an overall trajectory of outcomes, although a higher rate of completion at the 3 month followup would have added to the robustness of the results. Cronbach’s alpha values are reduced by low numbers of items within the questionnaire. It may be necessary to supplement the physical domain with additional items that address symptom-related concerns identified by patients to address the potential for measurement variance in the physical symptom subscale.

The usefulness of QoL measures is based on the principle that the subtleties of a patient’s health are multifaceted and are often difficult for healthcare professionals to access, whereas a more patient-centered perspective on QoL provides a more accurate assessment. Physical factors may not be the sole determining motivator for patients seeking intervention for their condition. Patients’ expectations of improvement shape their perceptions of QoL which, in turn is grounded on their re-engaging in psychologically meaningful activity [33, 34].

## Conclusions

The TASQ was developed based on information provided by a large cohort of AS patients and our person-centred approach to developing the TASQ highlighted the factors of QoL identified as most important to them. The subscales tap meaningful outcomes for this patient population, and the items are relevant and sensitive to changes in QoL following therapeutic intervention. In addition, the TASQ is a brief self-administered questionnaire that is easy to score, and thus may be clinically useful for monitoring QoL in patients with AS at various stages of their illness. Findings from our development and QoL studies suggest that the TASQ is valid and reliable and provides insight into the QoL goals that patients value most.

## Data Availability

The datasets generated and analyzed during the current study are not publically available due to permission for data sharing not obtained from participants at the time of recruitment to the study and participants being potentially identifiable.

## Abbreviations

AS: Aortic stenosis
EQ-5D: Euro quality of life 5 dimensions
IIRS: Illness intrusiveness rating scale
KCCQ: Kansas City cardiomyopathy questionnaire
MLHF: Minnesota living with heart failure
PROs: Patient reported outcomes
SF-36: Short form health survey – 36
TASQ: Toronto aortic stenosis quality of life questionnaire
TAVI: Transcatheter aortic valve implantation
